# Creation and manipulation of topological states in chiral nematic microspheres

**DOI:** 10.1038/ncomms8603

**Published:** 2015-07-06

**Authors:** Tetiana Orlova, Sarah Jane Aßhoff, Tadatsugu Yamaguchi, Nathalie Katsonis, Etienne Brasselet

**Affiliations:** 1 Laboratoire Ondes et Matière d'Aquitaine, University of Bordeaux, CNRS, 351 cours de la Libération, Talence F-33400, France; 2 Laboratory for Biomolecular Nanotechnology, MESA+ Institute for Nanotechnology, University of Twente, PO Box 207, Enschede 7500AE, The Netherlands

## Abstract

Topology is a universal concept that is encountered in daily life and is known to determine many static and dynamical properties of matter. Taming and controlling the topology of materials therefore constitutes a contemporary interdisciplinary challenge. Building on the controllable spatial properties of soft matter appears as a relevant strategy to address the challenge, in particular, because it may lead to paradigmatic model systems that allow checking theories experimentally. Here we report experimentally on a wealth of complex free-standing metastable topological architectures at the micron scale, in frustrated chiral nematic droplets. These results support recent works predicting the formation of free-standing knotted and linked disclination structures in confined chiral nematic fluids. We also demonstrate that various kinds of external fields (thermal, electrical and optical) can be used to achieve topological remote control. All this may foster the development of new devices based on topologically structured soft media.

## Abstract (web summary)

Controlling the topology of matter offers an alternative way to make functional materials, among which liquid crystals hold promise due to their spontaneously occurring defects. Here Orlova *et al*. demonstrate the formation and remote control of topological structures in frustrated cholesteric droplets.

## Graphical abstract

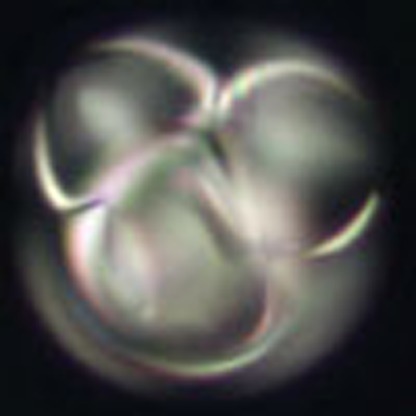

Topology usually refers to the description and classification of the spatial properties of material systems. In practice, the topological nature of a system can be viewed as the simplest way to describe it. Daily life examples are that of a simple cup with no handle, which is topologically equivalent to a sphere, whereas a mug with one handle belongs to the topology of a doughnut. Distinct topologies are usually associated with different kinds of defects that play an important role in many areas of science such as physics, chemistry and life sciences.

More specifically, soft condensed matter offers an interesting experimental playground and liquid crystal materials, which are well known to exhibit a wealth of spontaneously occurring defects and textures^1^, are a prime choice. Tailoring topological defects of liquid crystals is generally achieved by appropriate selection of the liquid crystal mesophase and orientational boundary conditions. A few years ago, complex three-dimensional networks of defect lines—knots and links—in the nematic order-parameter field have been experimentally unveiled in thin films of chiral nematics containing colloidal solid microspheres^2^,^3^ (see also ref. 4 for very recent developments). Then, the case of more complex solid micro-objects have been explored theoretically and experimentally, which includes the case of non-orientable ribbon-like colloids in chiral nematics^5^ as well as knotted rope-like colloids in non-chiral nematics^6^. Recalling de Gennes analogy between liquid crystals and magnetism and, on the other hand, the fact that liquid crystals are widely used in photonic technologies, these studies are likely to lay the foundations of future technologies based on the generation and control of arbitrarily complex topological circuitries.

A new twist has taken place recently by considering chiral nematic liquid crystals (cholesterics) confined in two dimensions (films) or three dimensions (droplets) without the need of colloidal particles. Cholesterics are usually prepared by adding a small fraction of a chiral dopant into an achiral nematic mesophase and are characterized by a helical order for the director that is a unit vector indicating the local average orientation of the molecules. A cholesteric is basically defined by its supramolecular helix pitch *p* that is the distance over which the director rotates by 2*π*, and the handedness of its intrinsic chirality. Actually, both two-dimensional and three-dimensional confinement of a cholesteric under perpendicular surface anchoring conditions can forbid the establishment of a continuous helical director field—a phenomenon often called frustration—thereby fostering the appearance of metastable topological defects. In particular, metastable particle-like topological structures have been predicted and observed in frustrated cholesteric films a few years ago^7^. Since then, the topological diversity of such systems has been explored and a variety of individual^8^ as well as linked and self-assembled^9^ structures have now been identified. On the other hand, in the case of frustrated cholesteric droplets, the existence of a rich variety of free-standing metastable topological structures has been numerically unveiled^10^, among which knots and links, which remain elusive so far.

Taming free-standing three-dimensional topological structures is a challenge that is relevant to numerous research fields. Creating and manipulating knots and links contributes to this multidisciplinary challenge, because these are generic features of topological manifestations. Efforts from other research fields involve the engineering and observation of vortex knots in optics^11^ and hydrodynamics^12^. Metastable knots in condensed matter come with an essential added value—the long-term preservation of the information—so that it can be read again by using appropriate control strategies. An important example is provided by localized topological excitations in magnetic materials that can be turned on and off^13^ and show great promise for the elaboration of future ultra-high density magnetic devices.

In this respect, ‘soft' alternatives are well-worth considering, as they combine remote-controlled features to topological diversity. Recently, the case of non-chiral nematics confined in non-trivial volumes with handles having perpendicular surface anchoring conditions^14^ has been experimentally considered. Surprisingly, there are only a few experimental studies of the three-dimensional ordering of chiral nematics confined in a spherical volume with perpendicular surface anchoring conditions. In 1973, Candau *et al*.^15^ reported on the observation of a three-dimensional radial distribution of the director when *p*>*R*, where *R* is the spherical droplet radius. On the other hand, when *p*<<*R*, they observed a radial distribution of the cholesteric helical axis with a radial disclination, similarly to the case of parallel surface anchoring^16^,^17^,^18^. Still, Candau *et al*. stressed that there should be additional defects to satisfy perpendicular boundary conditions, however without experimental support. In 1982, Kurik and Lavrentovich^19^ reported on the observation of an equatorial disclination and point surface singularity. For sufficiently small pitch, *p*<*R*/10, these authors also concluded that both perpendicular and parallel surface anchoring cases behave similarly, whereas for larger pitch values, *p*>*R*/5, a novel defect appears—an equatorial disclination—that can relax into a point surface defect *p*>*R*. In 1993, Kitzerow and Crooker^20^ reported on the observation of fingerprint patterns that consist of concentric, elliptical or parallel lines said to correspond to the same three-dimensional director field observed under different viewing angles.

In the context of such scarce experimental state of the art, we report on the observation and the manipulation of complex free-standing metastable topological architectures in frustrated cholesteric droplets. From the structural point of view, we experimentally unveil a rich structural topological diversity that support the static predictions made in ref. 10 and we bring evidence of novel topological structures for large values of the ratio *R*/*p*, which were not yet reported so far^10^. In addition, we propose and experimentally demonstrate various routes of contactless real-time reconfiguration of complex three-dimensional director fields by using thermal, electrical or optical external fields. As a result, we envision the development of new types of photonic elements based on structured soft media, for instance, high-dimensional rewritable topological memories or novel kinds of three-dimensional optical phase masks.

## Results

### Topological diversity

**Figure 1:**
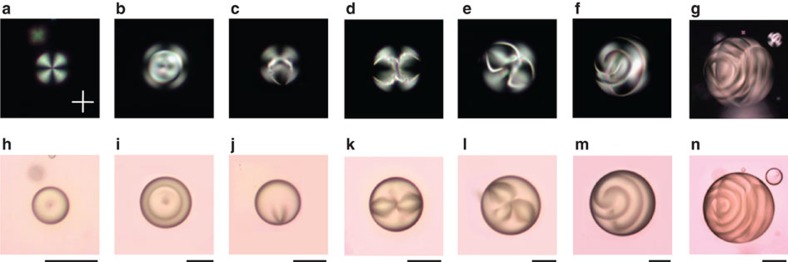
Topological diversity in frustrated cholesteric droplets. Examples of distincts metastable topological structures in cholesteric droplets with perpendicular surface anchoring observed between XPOL (upper row) and in FT (bottom row) under natural white light illumination. Experimental conditions: pitch *p*=55 μm and droplet diameter *d*=35 μm (**a**,**h**), *d*=96 μm (**b**,**i**), *d*=69 μm (**c**,**j**), *d*=80 μm (**d**,**k**), *d*=123 μm (**e**,**l**), *d*=167 μm (**f**,**m**) and *d*=271 μm (**g**,**n**), which correspond respectively to *N*=1.3, 3.5, 2.5, 2.9, 4.5, 6.1 and 9.8. White cross in **a** indicates the directions of the polarizers. Scale bars, 50 μm.

In a first set of experiments, we address the topological diversity issue by using cholesteric liquid crystal spherical droplets with perpendicular anchoring conditions immersed in a high-viscosity surrounding fluid (see Methods for preparation details). In practice, we proceed to the thermal quenching of the droplets by heating the sample above the nematic–isotropic transition temperature and then leaving the sample to relax at room temperature. Such a procedure corresponds to the experimental counterpart of the simulated temperature quench reported in ref. 10 to reach high-energy orientational configurations. The results are summarized in Fig. 1 for a pitch *p*=55 μm, where we introduce the dimensionless parameter *N*=2*d*/*p*, which represents the number of *π* turns of the director along the droplet diameter *d*. This facilitates the comparison with previous numerical predictions where *N* has been chosen as a control parameter to explore metastable states zoology^10^.

Hundreds of droplets with *d*≳10 μm (that is, *N*≳0.4) are observed using a microscope under natural white light illumination, either between crossed linear polarizers (XPOL) or in full transmission (FT). When *N* is sufficiently small, *N*≲1.2, XPOL images always exhibit a dark cross of extinction indicating a radial ordering of the director field. On the other hand, radial ordering can be observed for 1.2≲*N*≲1.4, whereas it is never observed for *N*≳1.4. An example of radially aligned cholesteric droplet is provided in Fig. 1a, which cannot be distinguished from the case of achiral nematic droplets with perpendicular anchoring conditions^15^,^21^. In particular, the presence of a hedgehog defect in the centre of radially ordered droplets is revealed by FT imaging as a dark point, see Fig. 1h.

This situation is the three-dimensional analogue of frustrated chiral nematic films whose helix can be unwound when *p* is sufficiently larger than the film thickness *d*, namely below the threshold value *N*_th_=*K*_3_/*K*_2_ (ref. 22), where *K*_2_ and *K*_3_ are the twist and bend Frank elastic constants of the liquid crystal. This typically gives *N*_th_≃2, whereas the bistability range typically extends down to *N*≃1.5 in the two-dimensional case^23^. Observed difference between the bistability ranges points out the possible role of the system dimensionality on the frustration phenomenon, though the role finite anchoring conditions and partial miscibility at the interface between the liquid crystal and the surrounding fluid cannot be discarded.

As *N* increases, distinct three-dimensional director topological configurations are observed, several of them being reported in Fig. 1. XPOL imaging is basically dedicated to the identification of smooth spatial variation of the optical anisotropy, whereas FT imaging is prone to reveal abrupt spatial variation of the refractive index. Therefore, their combination is useful towards the qualitative identification of complex director fields endowed with defect points and lines. For instance, a point-like defect surrounded by a loop of line defect is recognized from Fig. 1b,i, whereas the appearance of a point defect nearby the surface is inferred from Fig. 1c,j. More complex structures are also observed, see Fig. 1d,k and Fig. 1e,l, as well as spiralling disclinations, see Fig. 1f,m and Fig. 1g,n. Noticeably, the reminiscence of the dark cross of extinction in Fig. 1c–e indicates the presence of a radial `orientational background' that is however not a rule of thumb, as emphasized by Fig. 1b and Fig. 1f where the fourfold signature of the dark cross is only visible at the rim of the droplet, as expected from perpendicular surface anchoring conditions.

We also stress that every droplet configuration presented in Fig. 1 is not restricted to a given value but a range of *N*. For instance, observations similar to Fig. 1b,i are made in the typical range 2≲*N*≲6, whereas the cases shown in Fig. 1c,j and Fig. 1d,k are encountered for 2≲*N*≲4 and the one shown in Fig. 1e,l for 2≲*N*≲5. The spiralling structure shown in Fig. 1f,m are found over the range 3≲*N*≲7. This unambiguously demonstrates the multistable character of the metastable states regarding the geometrical parameter *N* and validates experimentally the theoretical finding that the number of distinct states primarily depends on *N* (ref. 10).

**Figure 2:**
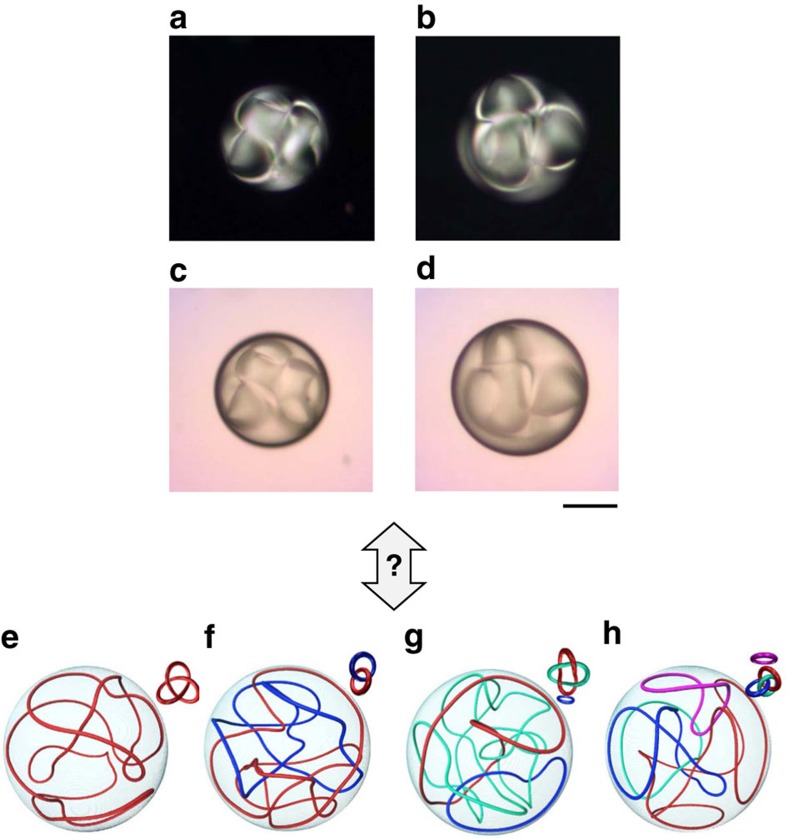
Complex metastable states. Examples of complex metastable topological structures in cholesteric droplets with perpendicular surface anchoring. Experimental conditions: pitch *p*=55 μm, droplet diameter *d*=105 μm (**a**,**c**) and *d*=125 μm (**b**,**d**) which correspond to *N*=3.8 and *N*=4.5. (**a**,**b**) XPOL imaging. (**c**,**d**) FT imaging. Scale bar, 50 μm. Bottom row: illustration of a few numerically predicted non-trivial topological states in ref. 10 when *N*=5, which consist of one (**e**) two (**f**) three (**g**) and four (**h**) disclination loops that are knotted or linked, as indicated in the insets. Sketches adapted from ref. 10.

Recent numerical predictions of a climax of topological complexity for intermediate values of *N*, especially with the emergence of knotted and linked director fields in the range 4≲*N*≲5 (ref. 10), call for experimental investigations. We find a few complex metastable states that are potential candidates. Two examples that corresponds to *N*=3.8 and *N*=4.5 are shown in Fig. 2. The visual inspection of Fig. 2a–d is likely to support the observation of non-trivial topological states. This is purposely illustrated by a question mark connection between our observations and a few examples of numerically predicted states in ref. 10, see Fig. 2e–h. Of course, a deeper insight should come from three-dimensional reconstruction of the director field, for instance via fluorescence confocal polarizing microscopy^24^ or non-linear microscopy^25^, which appears as a challenging experimental task.

Following ref. 10, another intriguing behaviour is predicted in the high-chirality regime. At *N*>>1, disclination lines lie nearby the surface of the droplet and organize into two-dimensional layered patterns that dictate the nature of the bulk director field. In particular, a uniformly spiralling disclination promotes a uniformly twisted director field whose qualitative understanding implies that the smectic-like subsurface disclination pattern screens the frustrating perpendicular surface anchoring conditions^10^. Remarkably, such a picture is consistent with the fact that the present case is the inverted analogue of solid colloids with perpendicular surface anchoring immersed in uniformly twisted cholesterics films theoretically analysed in ref. 26 and experimentally observed in ref. 3.

**Figure 3:**
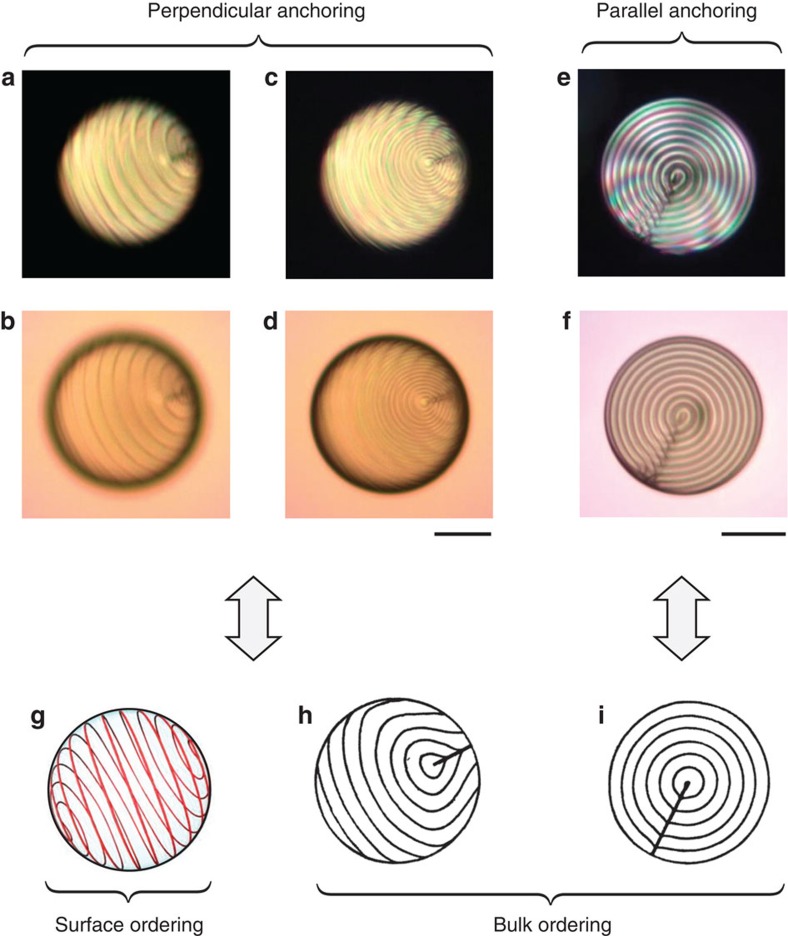
High-chirality regime. Typical XPOL (upper row) and FT (middle row) images of short-pitch cholesteric droplets with perpendicular (**a**–**d**) and parallel (**e**,**f**) surface anchoring. Perpendicular case: pitch *p*=12.3 μm, droplet diameter *d*=139 μm, *N*≈23. The two sets of pictures correspond to different imaging planes that allow to visualize the surface (**a**,**b**) and the equatorial plane (**c**,**d**) of the droplet. Parallel case: pitch *p*=12.3 μm, droplet diameter *d*=121 μm, *N*≈20. Scale bars, 50 μm. Bottom row: illustration of the director ordering. Perpendicular case: (**g**) shows typical subsurface spiralling disclination numerically predicted for short-pitch cholesterics in the case of droplets with perpendicular surface anchoring conditions, whereas **h** refers to bulk director field where cholesteric layers are indicated by parallel equidistant lines. Parallel case: **i** corresponds to well-known radial cholesteric droplet. Sketch **g** is adapted from ref. 10, whereas sketches **h**,**i** are adapted from ref. 27.

Here we report in Fig. 3 the typical situation encountered for short-pitch cholesterics, where panels (a,b) and (c,d) correspond to two different imaging planes at *N*≈23. More precisely, the surface of the droplet is brought into focus for the former XPOL and FT images, which clearly show a layer-like disclination pattern. On the other hand, the latter images refer to the droplet equatorial plane, for which the spiral is now out of focus. This experimentally demonstrates the surface character of the spiralling defect lines, whose ‘spiral pitch' Λ satisfies Λ≈*p* as expected theoretically^10^ and illustrated in Fig. 3g. However, the bulk ordering of the droplet differs from the predicted uniformly layered helical structure^10^. Instead, we observe an onion-like structure with a radial defect, see Fig. 3c,d, similar to the one observed for short-pitch cholesteric droplets with parallel surface anchoring^16^,^17^,^18^. This is emphasized in Fig. 3e,f where we present XPOL and FT images of a cholesteric droplet with parallel boundary conditions at *N*≈20 (see Methods for details on the preparation of the droplets), Also, it is instructive to put these observations closer to previously reported bulk structure of cholesteric droplets with parallel surface anchoring conditions^27^, see Fig. 3h,i. In fact, these results bring a direct proof that a screening mechanism is actually at work in the high-chirality regime, where the perpendicular surface anchoring conditions are replaced by effective parallel ones.

Importantly, we notice that all the structures presented in this section are stable in time. In practice, no structural evolution is observed at room temperature.

### Topological reconfiguration under thermal and electrical fields

As shown above, we observe of a wealth of metastable topological structures in cholesteric droplets with perpendicular surface anchoring of various diameters. Recalling that liquid crystals are well-known for their extreme sensitivity to external fields, the obtained topological structures are thus inherently stimuli responsive. To demonstrate experimentally the ability to reconfigure remotely, a given topological state is a necessary first step towards the development of topological memories, which implies writing and deleting topological information. Such a possibility is explored in the case of thermal and electrical fields by realizing two sets of independent experiments that consist in carrying out several thermal and electrical cycles on a given droplet.

**Figure 4:**
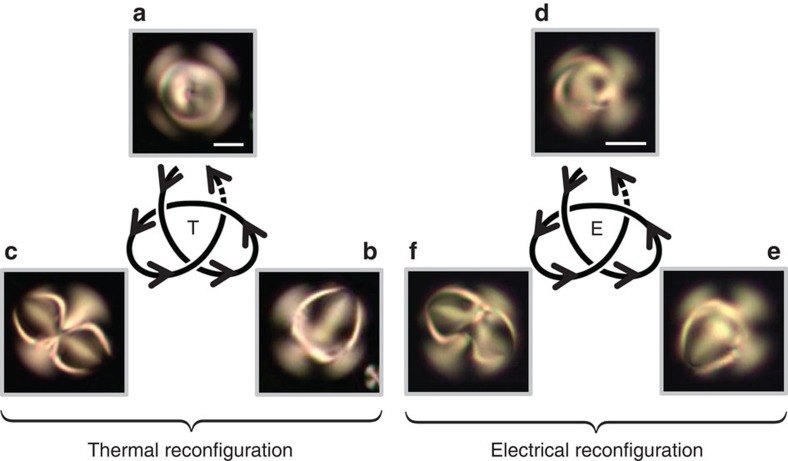
Thermally and electrically assisted topological reconfiguration at fixed pitch. Demonstration of topological transformations under the application of thermal (left part) and electrical (right part) fields for cholesteric droplets with perpendicular surface anchoring and pitch *p*=55 μm. All pictures correspond to XPOL imaging. Thermal case (see symbol ‘T'): droplet diameter *d*=85 μm, *N*=3.1. For each cycle, the liquid crystal is heated above the nematic–isotropic transition temperature and then cool down at room temperature, three distinct states are shown in **a**–**c**. Electrical case (see symbol ‘E'): droplet diameter *d*=67 μm, *N*=2.4. For each cycle, a 10-V_rms_ voltage at 2-kHz frequency is applied on the ≈250-μm-thick film in which the droplet is immersed, three distinct states are shown in **d**–**f**. Scale bars, 25 μm.

In the first case, the sample is heated above the clearing point and then cooled down to room temperature for 20 min. The three different topological states obtained following this procedure are shown in Fig. 4a–c. In the second case, an electrical field is abruptly applied on the droplet by imposing a 10-V_rms_ voltage at 2 kHz frequency on the ≈250-μm-thick film in which the droplet is immersed. After a few minutes, the voltage is turned off and the system is left to relax for 20 min. Three different topological states obtained following this procedure are shown in Fig. 4d–f.

In contrast to the thermal option that totally resets the liquid crystal ordering via nematic–isotropic phase transition, an electrical quench can be considered as a process that preserves the mean order parameter of the liquid crystal. Electrical reconfiguration thus demonstrates that the free energy barriers between topologically distinct metastable states can be easily reshaped by applying low-voltage electric fields. This may be useful towards the elaboration of remote-controlled high-dimensional topological liquid crystal memories. However, the present global control strategy does not yet allow switching between predetermined states, which remains an issue to be addressed in future work.

Still, it is worth mentioning that in the present study the observed patterns exhibit some robustness against the application of thermal or electrical fields, which emphasizes the metastable character of the topological structures. In experiments, topological transformations do not take place for moderate temperature elevation (typically up to 5° from the nematic–isotropic clearing point, which corresponds to a temperature elevation of a few degrees noting that room temperature is 22 °C in our case) or moderate applied voltage (typically below 5 V_rms_).

### Optomolecular control of topological transitions

Since the level of frustration of a cholesteric droplet with perpendicular boundary conditions is described by the diameter-to-pitch ratio *d*/*p*, molecular chirality can be considered as a control parameter to prepare tailored-made metastable states. Arguably, light-responsive cholesteric liquid crystals constitute an ideal system to investigate molecular control over topological transitions, as the photoinduced modifications of chirality have been shown to be controllable precisely^28^. Photoresponsive cholesterics are usually prepared by doping a nematic liquid crystal with a few per cent of chiral molecular photoswitches^28^,^29^. Here we choose light-driven molecular motors as dopants, because under irradiation with ultraviolet light, the handedness of these molecules is switched, but their overall shape is maintained^30^,^31^,^32^,^33^,^34^. For small concentrations of dopant, the pitch of the resulting cholesteric liquid crystal is completely determined by the structure of the dopant and by its concentration. Consequently, by adjusting the concentration of the molecular motor in the nematic liquid crystal, the chiral character of the system can be defined precisely.

Moreover, for these low levels of doping, it is known that the modification in elastic constants is negligible^30^,^35^. In particular, for the system we investigate here, it has been shown that assuming the same elastic constants for pure E7, and for E7 doped with molecular motors, theoretical models are in good agreement with experimental results^30^. Importantly, and in contrast to what happens when azobenzene-based molecules are used as photoswitchable dopants^36^,^37^, our system does not suffer from expulsion of the photoisomer towards the droplet boundaries under irradiation, because all isomers of these molecular motors show a high solubility in E7 (refs 30, 33, 38, 39).

Here the concentration of molecular motor **m**_1_ is tuned to reach the high-chirality regime (*N*>>1) in the initial state. Under irradiation with ultraviolet light, the chirality of the system is modified at the level of the molecular motor, and transmitted to the mesoscopic level (the cholesteric liquid crystal). Specifically, the motor has an overall helical shape that is right handed in the stable state, and left handed for its photoisomer. The ratio between these two forms determines the effective molecular chirality that is amplified by the cholesteric helix. Initially, the structural changes associated with the appearance of the photoisomerization of **m**_1_ express at the supramolecular level first as an increase of the cholesteric pitch. After a few minutes of irradiation the compensation point is reached, which corresponds to a pseudo-nematic liquid crystal mixture (*N*=0). Ultraviolet irradiation is then stopped. Importantly, it has been shown previously that under irradiation, the reorganization of the liquid crystal follows the kinetics of photoisomerization of the motor^30^. This means that, in principle, any value of the cholesteric pitch, hence *N*, can be reached by tuning the characteristics of irradiation. The photochemical reaction being fully reversible with molecular motor **m**_1_, thermal relaxation brings the system back to the initial state.

**Figure 5:**
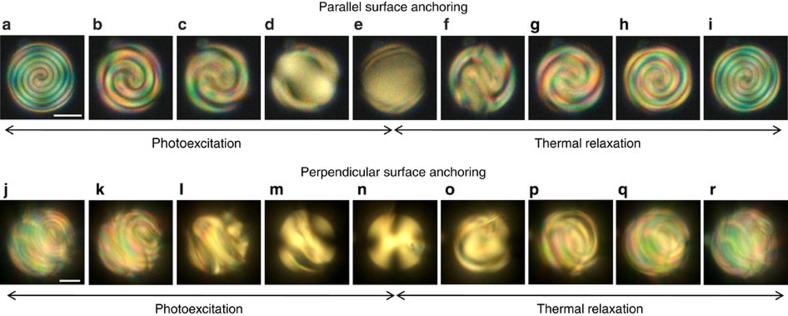
Optical chiral control. Light-induced structural changes followed by thermal relaxation of cholesteric droplets with photo-tunable pitch in the case of parallel (upper row) and perpendicular (bottom row) surface anchoring, using motor **m**_1_. All pictures correspond to XPOL imaging. Parallel case: droplet diameter *d*=24 μm, *N*≈12 before illumination is turned on. Illumination time is 0 s (**a**), 12.2 s (**b**), 14.4 s (**c**), 17.7 s (**d**) and 19.9 s (**e**), whereas relaxation time after ultraviolet radiation is turned off is 2.2 s (**f**), 7.7 s (**g**), 22.1 s (**h**) and 53 s (**i**). Perpendicular case: droplet diameter *d*=34 μm, *N*≈20 before illumination is turned on. Illumination time is 0 s (**j**), 20 s (**k**), 35.5 s (**l**), 47.6 s (**m**) and 163.7 s (**n**), whereas relaxation time after ultraviolet radiation is turned off is 15.5 s (**o**), 25.4 s (**p**), 40.9 s (**q**) and 171.3 s (**r**). Scale bars, 10 μm.

We first investigate the case of droplets with parallel surface anchoring to benchmark the optomolecular control of the director field structuring in spherical confinement. Indeed, the expected structural scenario as *d*/*p* is varied is well known in that case^17^ and correspond to an onion-like structure with a radial (or diametral) defect at large *N* as shown in Fig. 3e,f, whereas the director field tends to a bipolar structure as *N* goes to zero. Our proposition to vary the chirality parameter in a continuous manner thus offers an original opportunity to explore the structural bifurcation scenario for the three-dimensional director field. The results are shown in the upper part of Fig. 5 where the initial condition (Fig. 5a) corresponds to *N*≈12, noting that the viewing direction is here directed along the radial defect. As the ultraviolet irradiation is turned on, the pitch of the spiralling structure monotonously increases (Fig. 5b,c) to eventually disappear (Fig. 5d) and exhibit a bipolar configuration (Fig. 5e). When chirality almost vanishes, the ultraviolet illumination is turned off and the system is left to relax at room temperature and a reverse scenario is observed, see Fig. 5f–i. These results bring a vivid demonstration of three-dimensional photoinduced transformations, which extend previous results limited to tuning in the high-chirality regime^40^.

The case of photoresponsive droplets with perpendicular surface anchoring is shown in the bottom part of Fig. 5 where the initial condition (Fig. 5j) corresponds to *N*≈20 and exhibits a structure similar to the one presented in Fig. 3a–d. Drastic changes of the three-dimensional director field are observed as the pitch increases under ultraviolet illumination until the pseudo-nematic state is eventually reached (Fig. 5n). The observed structural scenario, however, does not give access to the broad spectrum of topological structures identified in thermal quench experiments (Figs 1 and 2), thereby pointing out a distinct exploration of the free energy landscape. Indeed, abrupt spatiotemporal changes of the order parameter occurring during a thermal quench possibly lead to any orientational state, whereas continuous variation of the pitch during the optomolecular process is related to smooth configuration changes.

Full exploration of the free energy landscape will consequently require the development of advanced control strategies. However, the stability of the target structure and the kinetics of the configurational changes can be controlled by tuning the power of irradiation and/or the kinetics of relaxation of the dopant through molecular design. Indeed, it is known that the efficiency of the photostep depends on the electronic structure of the overcrowded double bond and that the efficiency of molecular motors can be controlled optically by using pulsed light^41^. Independently, the kinetics of the thermal relaxation of molecular motors are determined by the transition state energies. By expanding the upper half of the motor, contracting the overcrowded double bond or by decreasing the steric hindrance of the substituents in the upper half, the relaxation is slowed down significantly and the photoisomer is stabilized^42^. Moreover, as the reorganization of the nematic host is much faster than the isomerization of the motor, the kinetics of texture changes in the droplets are fully determined by the kinetics of the molecular motor^30^,^33^. Consequently, molecular design gives the freedom to adjust the kinetics of the system depending on the target application.

**Figure 6:**
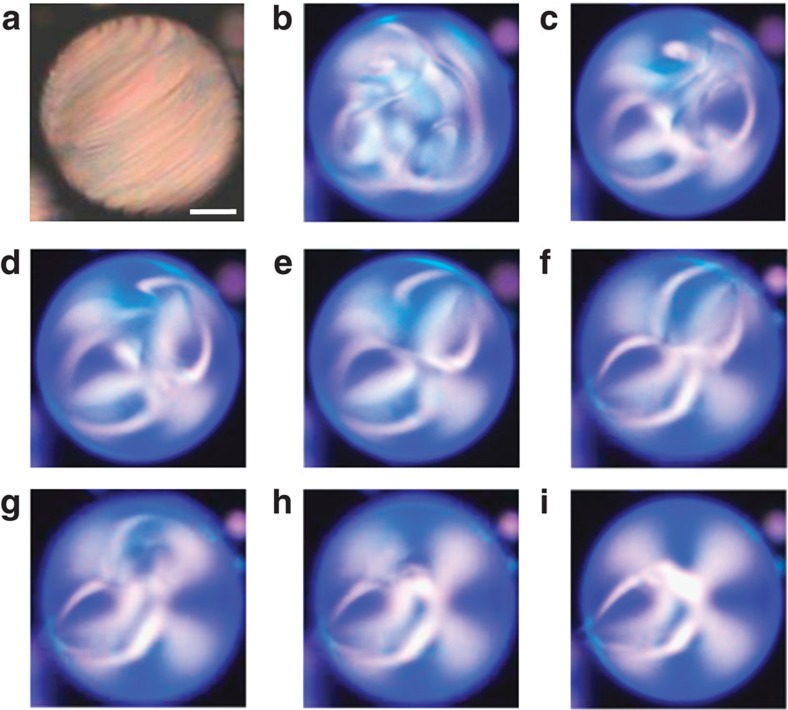
Photoinduced topological transitions. XPOL imaging observation of light-induced structural change for a photoactive cholesteric droplet with perpendicular surface anchoring, using motor **m**_2_. Droplet diameter is *d*≈ 90 μm and *N*≈40 before ultraviolet illumination is turned on, see **a**. Transition from complex (**b**) to much simpler (**i**) topological state takes place as irradiation time *t* increases: *t*=339.2 s (**b**), *t*=473 s (**c**), *t*=486.2 s (**d**), *t*=496.2 s (**e**), *t*=595.6 s (**f**), *t*=639.8 s (**g**), *t*=646.5 s (**h**) and *t*=647.6 s (**i**). Presence of blue colour on images **b**–**i** is due to high ultraviolet light intensity. Scale bar, 20 μm.

We also explore topological transformations under chiroptical molecular control by using another molecular motor **m**_2_ (see Methods) in order to demonstrate the generic character of the approach we propose. The motor **m**_2_ is characterized by a longer half-life time (about three times longer than that of **m**_1_) that leads to a long-term stability of the topological pattern obtained in the photostationary state (shown in Fig. 6i). Moreover, different irradiation conditions for MIX1 and MIX2 droplets allow to gain more intermediate patterns during photoinduced transformation of MIX2 droplets. In particular, as shown in Fig. 6, we observe well-defined transformations between states belonging to the topological zoo presented in Fig. 1 for passive chiral nematic droplets.

## Discussion

Experimental demonstration of both metastable and multistable complex topological states in chiral nematic spherical droplets with perpendicular boundary conditions is reported. Among the unveiled three-dimensional defect structures, highly complex ones are likely to qualify as free-standing knotted and/or linked director fields. Although direct three-dimensional determination of the disclination lines entanglement remains a necessary experimental step to cross, our results support recent predictions stressing that the interplay between confinement and chirality in spherical geometry may lead to high level of topological diversity^10^. Present work and the one reported in ref. 14, that deals with the interplay between confinement and non-trivial geometries in the absence of chirality, thus complement one another.

The extreme sensitivity of liquid crystal systems under external fields (for example, thermal, electrical, magnetic or optical ones) brings several advantages. First, this can be useful to proceed to knot simplification to ease experimental identification, for instance, by applying smooth global perturbation acting as topology-preserving Reidemeister moves, though keeping in mind that high-amplitude perturbation can induce topological transformation. Second, high-dimensional rewritable topological memories can be envisioned. In that case, the main challenge is the deterministic topological engineering and the reported signatures of complex free energy landscape associated with the three-dimensional director field imply challenging future experimental developments. In particular, the use of tightly focused laser beams is an attractive option to apply local perturbations, as demonstrated in the case of the inverse problem that consists of solid colloids embedded in chiral nematic films^2^. In this context, the optomolecular control of the intrinsic chirality of the material at ultra-low optical power^43^, here restricted to global variation of the cholesteric pitch, is a promising option.

Another issue raised by the present work concerns the bulk structuring in the high-chirality regime. Indeed, although predicted subsurfaces disclination lines presenting a smectic-like order^10^ are experimentally confirmed, the expected uniformly twisted cholesteric bulk ordering is not found. Instead, onion-like structure having a radial defect is observed. The similarity of the latter director field with that of cholesteric droplets with parallel surface anchoring stresses the screening effect of the subsurface defect structure unveiled in ref. 10, which provide a droplet with perpendicular surface anchoring with an effective parallel one. Our experiment of photoinduced topological transformations under varying pitch thus appears as the chiral analogue of the topological dynamics of defects reported in ref. 44 in the case of achiral nematic droplets with boundary conditions varying from perpendicular to parallel and vice versa.

Finally, these results are relevant in terms of shaping light fields also, and could thus set the basis for optical reading of topological memories. Moreover, our results could promote the development of enhanced spin–orbit topological shaping of light by using with liquid crystal defects, an approach that has been limited so far to optical vortex generation by using either hedgehog^45^, umbilical^46^ or Schlieren^47^ defects in nematics, localized defect structures in cholesterics^48^ and focal conic domains in smectics^49^.

## Methods

### Preparation of passive cholesteric droplets

Cholesteric droplets with perpendicular surface anchoring conditions are obtained by dispersing the material in high-viscosity (300000 cSt) polydimethylsiloxane (from ABCR), whereas parallel surface anchoring conditions are obtained by dispersion in glycerol. Two chiral nematic liquid crystal mixtures have been used, with cholesteric pitch *p*=12.3 and 55 μm, made of the nematic 5CB doped with S-811 (both from Merck). The pitch values are measured from Cano wedge technique. Droplets are observed using Olympus IX71 polarized optical microscope not less than 30 min after sample preparation. Images are recorded with a DCC 1645C Thorlabs camera. Heating of the samples is performed using HI-55Dp heater (from Cell MicroControls) and liquid crystal controller LCC25 (from Thorlabs) is used for electric field application.

### Preparation of photoactive cholesteric droplets

A photoresponsive cholesteric liquid crystal MIX1 is prepared by dissolving 1 wt% of light-driven molecular motor **m**_1_ (2((2S)-(P)-4-fluoren-9-ylidene-3-methyl-1,2,3,4-tetrahydro-phenanthrene)) in dichloromethane and then added to the nematic liquid crystal mixture E7 (from Merck). Molecular motor **m**_1_ is characterized by a half-life time of *τ*_1_=190 s in hexane^32^. Similarly, MIX2 is prepared by dissolving 0.34 wt% of light-driven molecular motor **m**_2_ ((R)-9-(2-methyl-2,3-dihydro-1H-cyclopenta[a]naphtalen-1-ylidene)-9H-fluorene) and 0.03 wt% of shape-persistent chiral dopant ((R)-2,2′-(1,3-Propylenedioxy)-1,1′-binaphthalene) in dichloromethane and then added to E7. The characteristic half-time of **m**_2_ is *τ*_2_=580 s in hexane^32^. The cholesterics MIX1 and MIX2 are ready for use after the solvent is evaporated at 40 °C under a stream of nitrogen. For MIX1 microspheres, perpendicular surface anchoring is ensured by using a 5-mM solution of SDS in MiliQ water, whereas a 4-wt% PVA solution in 1:1 mixture of MiliQ water and glycerol is used in the parallel case. For MIX2 microspheres, perpendicular surface anchoring is ensured by using polydimethylsiloxane. For homeotropic droplets, the use of both SDS and PDMS lead to observing a black cross between crossed-polarizers for small enough *N* values, which indicates strong perpendicular boundary conditions. For the droplets in glycerol, the well-known radial texture was observed for large enough *N* values, which constitutes a clear signature for planar anchoring.

### Optical control of photoactive cholesteric droplets

Ultraviolet irradiation is achieved using a Hönle bluepoint LED lamp (*λ*=365 nm). MIX1 droplets with parallel surface anchoring are irradiated at intensity ≈50 mW cm^−2^, whereas MIX1 droplets with perpendicular surface anchoring are irradiated at intensity ≈180 mW cm^−2^. MIX2 droplets are irradiated with an intensity of ≈340 mW cm^−2^. Droplets are observed using Olympus BX51 polarized optical microscope and images are recorded by DP73 Olympus camera. The value of *N* is evaluated from crossed-polarized images before the ultraviolet light is turned on from the relationship *N*=*d*/*δ* where *δ* is the spatial period of the bulk onion-like structure at *t*=0 s, see Fig. 5a for the parallel case and Fig. 5j for the perpendicular case.

## Additional information

**How to cite this article:** Orlova, T. *et al*. Creation and manipulation of topological states in chiral nematic microspheres. *Nat. Commun.* 6:7603 doi: 10.1038/ncomms8603 (2015).

## References

[b1] Dierking I. Textures of liquid crystals Wiley-VCH (2003).

[b2] Tkalec U., Ravnik M., Čopar S., Žumer S. & Muševič I. Reconfigurable knots and links in chiral nematic colloids. Science 333, 62–65 (2011). 21719671 10.1126/science.1205705

[b3] Jampani V. . Colloidal entanglement in highly twisted chiral nematic colloids: twisted loops, hopf links, and trefoil knots. Phys. Rev. E 84, 031703 (2011). 10.1103/PhysRevE.84.031703 22060386

[b4] Čopar S., Tkalec U., Muševič I. & Žumer S. Knot theory realizations in nematic colloids. Proc. Natl Acad. Sci. USA 112, 1675–1680 (2015). 25624467 10.1073/pnas.1417178112 PMC4330751

[b5] Machon T. & Alexander G. P. Knots and nonorientable surfaces in chiral nematics. Proc. Natl Acad. Sci. USA 110, 14174–14179 (2013). 23940365 10.1073/pnas.1308225110 PMC3761586

[b6] Martinez A. . Mutually tangled colloidal knots and induced defect loops in nematic fields. Nat. Mater. 13, 258–263 (2014). 24390381 10.1038/nmat3840

[b7] Smalyukh I. I., Lansac Y., Clark N. A. & Trivedi R. P. Three-dimensional structure and multistable optical switching of triple-twisted particle-like excitations in anisotropic fluids. Nat. Mater. 9, 139–145 (2010). 19966792 10.1038/nmat2592

[b8] Ackerman P. J., Trivedi R. P., Senyuk B., van de Lagemaat J. & Smalyukh I. I. Two-dimensional skyrmions and other solitonic structures in confinement-frustrated chiral nematics. Phys. Rev. E 90, 012505 (2014). 10.1103/PhysRevE.90.012505 25122322

[b9] Loussert C. & Brasselet E. Multiple chiral topological states in liquid crystals from unstructured light beams. Appl. Phys. Lett. 104, 051911 (2014).

[b10] Seč D., Čopar S. & Žumer S. Topological zoo of free-standing knots in confined chiral nematic fluids. Nat. Commun. 5, 3057 (2014). 24419153 10.1038/ncomms4057

[b11] Dennis M. R., King R. P., Jack B., O'Holleran K. & Padgett M. J. Isolated optical vortex knots. Nat. Phys. 6, 118–121 (2010).

[b12] Kleckner D. & Irvine W. T. M. Creation and dynamics of knotted vortices. Nat. Phys. 9, 253–258 (2013).

[b13] Romming N. . Writing and deleting single magnetic skyrmions. Science 341, 636–639 (2013). 23929977 10.1126/science.1240573

[b14] Tasinkevych M., Campbell M. G. & Smalyukh I. I. Splitting, linking, knotting, and solitonic escape of topological defects in nematic drops with handles. Proc. Natl Acad. Sci. USA 111, 16268–16273 (2014). 25369931 10.1073/pnas.1405928111 PMC4246280

[b15] Candau S., Roy P. L. & Debeauvais F. Magnetic field effects in nematic and cholesteric droplets suspended in a isotropic liquid. Mol. Cryst. Liq. Cryst. 23, 283–297 (1973).

[b16] Bezić J. & Žumer S. Structures of the cholesteric liquid crystal droplets with parallel surface anchoring. Liq. Cryst. 11, 593–619 (1992).

[b17] Xu F. & Crooker P. Chiral nematic droplets with parallel surface anchoring. Phys. Rev. E 56, 6853–6860 (1997).

[b18] Seč D., Porenta T., Ravnik M. & Žumer S. Geometrical frustration of chiral ordering in cholesteric droplets. Soft Matter 8, 11982–11988 (2012).

[b19] Kurik M. & Lavrentovich O. Negative-positive monopole transitions in cholesteric liquid crystals. JETP Lett. 35, 444–447 (1982).

[b20] Kitzerow H.-S. & Crooker P. Electric field effects on the droplet structure in polymer dispersed cholesteric liquid crystals. Liq. Cryst. 13, 31–43 (1993).

[b21] Dubois-Violette E. & Parodi O. Emulsions nématiques. effets de champ magnétiques et effet piézoélectriques. J. Phys. Colloques 30, 57–64 (1969).

[b22] Zel'dovich B. Y. & Tabiryan N. V. Fréedericksz transition in cholesteric liquid crystal without external fields. JETP Lett. 34, 406–408 (1981).

[b23] Pirkl S., Ribière P. & Oswald P. Forming process and stability of bubble domains in dielectrically positive cholesteric liquid crystals. Liq. Cryst. 13, 413–425 (1993).

[b24] Smalyukh I. I., Shiyanovskii S. V. & Lavrentovich O. D. Three-dimensional imaging of orientational order by fluorescence confocal polarizing microscopy. Chem. Phys. Lett. 336, 88–96 (2001).

[b25] Trivedi R., Lee T., Bertness K. A. & Smalyukh I. I. Three dimensional optical manipulation and structural imaging of soft materials by use of laser tweezers and multimodal nonlinear microscopy. Opt. Express 18, 27658–27669 (2010). 21197040 10.1364/OE.18.027658

[b26] Lintuvuori J. S., Marenduzzo D., Stratford K. & Cates M. E. Colloids in liquid crystals: a lattice boltzmann study. J. Mat. Chem. 20, 10547–10552 (2010).

[b27] Bouligand Y. & Livolant F. The organization of cholesteric spherulites. J. Phys. 45, 1899–1923 (1984).

[b28] Katsonis N., Lacaze E. & Ferrarini A. Controlling chirality with macroscopic helix inversion in cholesteric liquid crystals. J. Mater. Chem. 22, 7088–7097 (2012).

[b29] Broer D. J., Crawford G. P. & Žumer S. Cross-Linked Liquid Crystalline Systems. From Rigid Polymer Networks to Elastomers CRC Press (2011).

[b30] Bosco A. . Photoinduced reorganization of motor-doped chiral liquid crystals: bridging molecular isomerization and texture rotation. J. Am. Chem. Soc. 130, 14615–14624 (2008). 18839947 10.1021/ja8039629

[b31] Vicario J., Meetsma A. & Feringa B. L. Controlling the speed of rotation in molecular motors. dramatic acceleration of the rotary motion by structural modification. Chem. Commun. 47, 5910–5912 (2005). 10.1039/b507264f 16317472

[b32] Vicario J., Walko M., Meetsma A. & Feringa B. L. Fine tuning of the rotary motion by structural modification in light-driven unidirectional molecular motors. J. Am. Chem. Soc. 128, 5127–5135 (2006). 16608348 10.1021/ja058303m

[b33] Aßhoff S. J. . Time-programmed helix inversion in phototunable liquid crystals. Chem. Commun. 49, 4256–4258 (2013). 10.1039/c2cc37161h 23230570

[b34] Katsonis N., Lubomska M., Pollard M., Feringa B. & Rudolf P. Synthetic light-activated molecular switches and motors on surfaces. Prog. Surface Sci. 82, 407–434 (2007).

[b35] Grebyonkin M., Beresnev G. & Belyaev V. Visco-elastic properties of liquid crystalline mixtures. Mol. Cryst. Liq. Cryst. 103, 1–18 (1983).

[b36] Aronzon D. . Trans-cis isomerization of an azoxybenzene liquid crystal. Liq. Cryst. 34, 707–718 (2007).

[b37] Dubtsov A., Pasechnik S., Shmeliova D. & Kralj S. Light and phospholipid driven structural transitions in nematic microdroplets. Appl. Phys. Lett. 105, 151606 (2014).

[b38] Chen J. . Textures of cholesteric droplets controlled by photo-switching chirality at the molecular level. J. Mater. Chem. C 2, 8137–8141 (2014).

[b39] Eelkema R. . Molecular machines: nanomotor rotates microscale objects. Nature 440, 163 (2006). 16525460 10.1038/440163a

[b40] Lin J.-D. . Optically tunable/switchable omnidirectionally spherical microlaser based on a dye-doped cholesteric liquid crystal microdroplet with an azo-chiral dopant. Opt. Express 21, 15765–15776 (2013). 23842363 10.1364/OE.21.015765

[b41] Conyard J. . Ultrafast dynamics in the power stroke of a molecular rotary motor. Nat. Chem. 4, 547–551 (2012). 22717439 10.1038/nchem.1343

[b42] Pollard M. M., Klok M., Pijper D. & Feringa B. L. Rate acceleration of light-driven rotary molecular motors. Adv. Funct. Mater. 17, 718–729 (2007).

[b43] Loussert C., Iamsaard S., Katsonis N. & Brasselet E. Subnanowatt opto-molecular generation of localized defects in chiral liquid crystals. Adv. Mater. 26, 4242–4246 (2014). 24789629 10.1002/adma.201400811

[b44] Volovik G. E. & Lavrentovich O. D. Topological dynamics of defects: boojums in nematic drops. Sov. Phys. JETP 58, 1159–1166 (1983).

[b45] Brasselet E., Murazawa N., Misawa H. & Juodkazis S. Optical vortices from liquid crystal droplets. Phys. Rev. Lett. 103, 103903 (2009). 19792312 10.1103/PhysRevLett.103.103903

[b46] Brasselet E. & Loussert C. Electrically controlled topological defects in liquid crystals as tunable spin-orbit encoders for photons. Opt. Lett. 36, 719–721 (2011). 21368960 10.1364/OL.36.000719

[b47] Loussert C., Delabre U. & Brasselet E. Manipulating the orbital angular momentum of light at the micron scale with nematic disclinations in a liquid crystal film. Phys. Rev. Lett. 111, 037802 (2013). 23909361 10.1103/PhysRevLett.111.037802

[b48] Yang B. & Brasselet E. Arbitrary vortex arrays realized from optical winding of frustrated chiral liquid crystals. J. Opt. 15, 044021 (2013).

[b49] Son B. . Optical vortex arrays from smectic liquid crystals. Opt. Express 22, 4699–4704 (2014). 24663788 10.1364/OE.22.004699

